# Large scale proteomic studies create novel privacy considerations

**DOI:** 10.1038/s41598-023-34866-6

**Published:** 2023-06-07

**Authors:** Andrew C. Hill, Claire Guo, Elizabeth M. Litkowski, Ani W. Manichaikul, Bing Yu, Iain R. Konigsberg, Betty A. Gorbet, Leslie A. Lange, Katherine A. Pratte, Katerina J. Kechris, Matthew DeCamp, Marilyn Coors, Victor E. Ortega, Stephen S. Rich, Jerome I. Rotter, Robert E. Gerzsten, Clary B. Clish, Jeffrey L. Curtis, Xiaowei Hu, Ma-en Obeidat, Melody Morris, Joseph Loureiro, Debby Ngo, Wanda K. O’Neal, Deborah A. Meyers, Eugene R. Bleecker, Brian D. Hobbs, Michael H. Cho, Farnoush Banaei-Kashani, Russell P. Bowler

**Affiliations:** 1 National Jewish Health, Denver, CO USA; 2 Colorado School of Public Health, Fort Collins, CO USA; 3 Center for Public Health Genomics, University of Virginia, Charlottesville, VA USA; 4 Department of Epidemiology and Human Genetics Center, UTHealth School of Public Health, Houston, TX USA; 5 University of Colorado – Anschutz Medical Campus, Aurora, CO USA; 6 Mayo Clinic, Rochester, MN USA; 7 Department of Pediatrics, The Institute for Translational Genomics and Population Sciences, The Lundquist Institute for Biomedical Innovation at Harbor-UCLA Medical Center, Torrance, CA USA; 8 Division of Cardiovascular Medicine, Cardiovascular Research Center, Beth Israel Deaconess Medical Center, Boston, MA USA; 9 Metabolomics Platform, Broad Institute of Massachusetts Institute of Technology and Harvard, Cambridge, MA USA; 10 University of Michigan, Ann Arbor, MI USA; 11 Novartis, Basel, Switzerland; 12 University of North Carolina at Chapel Hill, Chapel Hill, NC USA; 13 University of Arizona, Tucson, AZ USA; 14 Harvard Medical School, Boston, MA USA; 15 Division of Pulmonary and Critical Care Medicine, Brigham and Women’s Hospital, Boston, MA USA; 16 Channing Division of Network Medicine, Brigham and Women’s Hospital, Boston, MA USA; 17 University of Colorado Denver, Denver, CO USA

**Keywords:** Computational biology and bioinformatics, Databases, Proteome informatics, Statistical methods, Diagnostic markers

## Abstract

Privacy protection is a core principle of genomic but not proteomic research. We identified independent single nucleotide polymorphism (SNP) quantitative trait loci (pQTL) from COPDGene and Jackson Heart Study (JHS), calculated continuous protein level genotype probabilities, and then applied a naïve Bayesian approach to link SomaScan 1.3K proteomes to genomes for 2812 independent subjects from COPDGene, JHS, SubPopulations and InteRmediate Outcome Measures In COPD Study (SPIROMICS) and Multi-Ethnic Study of Atherosclerosis (MESA). We correctly linked 90–95% of proteomes to their correct genome and for 95–99% we identify the 1% most likely links. The linking accuracy in subjects with African ancestry was lower (~ 60%) unless training included diverse subjects. With larger profiling (SomaScan 5K) in the Atherosclerosis Risk Communities (ARIC) correct identification was > 99% even in mixed ancestry populations. We also linked proteomes-to-proteomes and used the proteome only to determine features such as sex, ancestry, and first-degree relatives. When serial proteomes are available, the linking algorithm can be used to identify and correct mislabeled samples. This work also demonstrates the importance of including diverse populations in omics research and that large proteomic datasets (> 1000 proteins) can be accurately linked to a specific genome through pQTL knowledge and should not be considered unidentifiable.

## Introduction

Nearly four decades ago Jeffreys et al.^1^ recognized that patterns of simple tandem-repetitive regions of DNA were specific for individuals and could be used for identifying specific individuals or close relatives. Although initially controversial, the DNA-fingerprinting technique was rapidly and widely adapted by forensic scientists and within a decade was in the public’s vernacular. Soon thereafter the results of the Human Genome Project were published^2,3^ and it is now recognized that there are millions of single nucleotide polymorphisms (SNP) which can distinguish individuals within large populations. Identifying individuals by genomics is a rising concern in research because advances in genotyping and sequencing have resulted in large genetic databases (dbGaP; GEO; EMBL-EBI) for both research and commercial use. The existence of newer genotyping technologies and large genomic databases has created concerns among policy makers regarding discrimination in health insurance and employment and resulted in new laws that address genetic information (e.g., the Genetic Information Non-discrimination Act of 2008) as well as privacy protection efforts such as the Global Alliance for Genomics and Health, which has created frameworks to ensure responsible and secure sharing of genomic and health-related data. A key feature of these policies in the United States is that they explicitly addressed genomic (single nucleotide, sequence, transcriptome, epigenomic, and gene expression) data only. Despite these policies, there have been multiple instances of “deidentified” personal information linked back to individual genetic profiles^4^, including well publicized individuals such as Henrietta Lacks^5^. There have also been methods proposed which can link expression data to genotype through eQTLs^6^.

Although lagging behind genotype and sequencing advances by 5–10 years, exponential technological advances in high throughput proteomics are leading to the creation of similar large databases with sensitive personal information. Concurrently there are studies which demonstrate that many proteins^7,8^ have genetic quantitative trait loci (QTLs), but current practice is to consider these datasets as deidentified data. In this manuscript we show that even limited proteome profiles without peptide sequencing can be linked to specific individuals by using prior independent knowledge of these QTLs and we provide a bioinformatic solution which obfuscates reidentification, yet still preserves at least some biomarker-phenotype relationships. These findings suggest an immediate need to change policy regarding non-genomic data used for research or commercial use.

## Methods

### Study populations

All study participants provided written informed consent approved by institutional review boards (IRBs). COPDGene and Jackson Heart Study (JHS) cohorts were randomly split into training and testing datasets and training subjects were not included in the testing cohort. Other independent cohorts used for testing included Subpopulations and Intermediate Outcome Measures in COPD Study (SPIROMICS) and Multi-Ethnic Study of Atherosclerosis (MESA). Race was self-reported. Characteristics of subjects used for training and test are shown below with summary demographics in Table 1. This manuscript was approved by the publication committees of the cohorts listed below as well as the NHLBI Trans-Omics for Precision Medicine (TOPMed). All research was performed in accordance with relevant guidelines/regulations and informed consent was obtained from all participants and/or their legal guardians. Research involving human research participants was performed in accordance with the Declaration of Helsinki.

**Table 1:** Characteristics of training cohort and independent testing cohorts with SomaScan 1.3K.

Cohort	Training		Testing			
	COPDGene	JHS	SPIROMICS	COPDGene	JHS	MESA
Proteomes	1184	1028	258	547	1027	948
Genomes			2638	9970	3406	5308
Gender (% female)	50.1%	60.4%	46.5%	46.6%	61.6%	52.8%
Age (± SD)	61.6 ± 9.1	56.0 ± 12.9	60.0 ± 9.2	67.9 ± 8.5	55.2 ± 12.6	60.7 ± 9.7
**Race/ethnicity (self-reported)**						
White, non-Hispanic	87%	–	71%	91%	–	35%
Black, non-Hispanic	13%	100%	23%	9%	100%	34%
Asian, non-Hispanic	–	–	5%	–	–	11%
Hispanic	–	–		–	–	19%

*SD* standard deviation, *SPIROMICS* SubPopulations and InteRmediate Outcome Measures In COPD Study, *JHS* Jackson Heart Study, *MESA* Multi-Ethnic Study of Atherosclerosis.

#### COPDGene

The NIH-sponsored multicenter Genetic Epidemiology of COPD (COPDGene (ClinicalTrials.gov Identifier: NCT01969344)) enrolled 10,263 non-Hispanic white (NHW) and Black (AA) individuals from January 2008 until April 2011 (Phase 1) who were aged 45–80 with ≥ 10 pack-year smoking history and no exacerbations for > 30 days and 457 age and gender matched healthy individuals with no history of smoking were enrolled as controls^9^. Subjects were genotyped using an Illumina HumanOmni Express^10^. 1184 subjects from the enrollment visit (P1) participated in an ancillary study in which they provided p100 (BD) fresh frozen plasma used for SomaScan 1.3K proteomic profiling which measured 1305 proteins. An additional 547 independent subjects, who only had SomaScan profiling at a 5-year follow up visit (P2) and not used in the training dataset, were used as an independent testing cohort. 5292 also had SomaScan 5K (v4.0) proteomes using plasma from a P2 visit and were randomly split into training and testing to assess whether scaling improved identification accuracy. COPDGene has been approved by the BRANY IRB.

#### Jackson Heart Study (JHS)

The NIH-sponsored (ClinicalTrials.gov Identifier: NCT00005485) enrolled 5306 African American residents living in the Jackson, MS, metropolitan statistical area (MSA) of Hinds, Madison, and Rankin Counties. 2055 gave consent for genetic research and also had SomaScan 1.3K proteomic profiling. Genotypes were extracted using TOPMed whole genome sequencing Freeze 8 to create a synthetic Illumina HumanOmniExpress genotype panel. The Jackson Heart Study (JHS) Institutional Review Board (IRB) Working Group (WG) is responsible for Overseeing and monitoring all JHS Institutional Review Board (IRB) activities Facilitating collaborative communications and transfer of information among the IRBs regulating the JHS: Jackson State University, University of Mississippi Medical Center, and Tougaloo College.

#### SPIROMICS

The NIH-sponsored Subpopulations and Intermediate Outcome Measures in COPD study (SPIROMICS) study (ClinicalTrials.gov Identifier: NCT01969344)^11^ enrolled 2984 subjects who were genotyped using the Illumina HumanOmniExpress genotyping platform^12^ of which 258 subjects underwent SomaScan 1.3K proteomic profiling using Visit 1 plasma. Additional SomaScan 7K data (version 4.1) were available for 2401 subjects from visit 1, 2, 4, and 5 (5132 total samples with proteomes). SPIROMICS has been approved by by the IRB at the University of North Carolina at Chapel Hill.

#### MESA

The NIH-sponsored Multi-Ethnic Study of Atherosclerosis (MESA) study (ClinicalTrials.gov Identifier: NCT00005487) recruited 6418 participants from four race/ethnic groups: Caucasian, African American, Hispanic, and Chinese. Whole genome sequencing (WGS) was performed at the Broad Institute of MIT and Harvard. SomaScan proteomics 1.3K profiling was performed at the Broad Institute and Beth Israel Proteomics Platform (HHSN268201600034I). The MESA study was approved by its six participating IRBs (see^13^) which include University of Washington, University of Vermont (biospecimen repository), Columbia University, Johns Hopkins University, Northwestern University, University of California, Los Angeles, University of Minnesota, and Wake Forest University.

#### ARIC

The Atherosclerosis Risk in Communities (ARIC) study initially enrolled 15,792 participants aged 45–64 years at four study centers in the United States: Washington County, MD; Forsyth County, NC; northwestern suburbs of Minneapolis, MN; and Jackson, MS between 1987 and 1989, aiming to investigate cardiovascular disease and its risk factors. Participants have undergone nine clinical visits. For current analysis, proteomic profiles were obtained from SomaLogic, via SomaScan 5K (v4.0) assay using freshly frozen blood plasma collected at ARIC visit 2 (1990–1992). Genotyping was performed using Affymetrix 6.0 array and imputed using TOPMed Freeze 5b datasets Details of genotyping and imputation quality control methods were previously described^14^. 242 out of 250 selected SNPs were obtained in both race groups, of which 176 were imputed in AA and 175 were imputed in EA. There were 2874 AAs and 9345 EA that genotypes available, therefore were included in the prediction analyses. The ARIC study has been approved by Institutional Review Boards (IRB) at all participating institutions: University of North Carolina at Chapel Hill IRB, Johns Hopkins University IRB, University of Minnesota IRB, and University of Mississippi Medical Center IRB.

### Proteome profiling

Proteomic profiles for 1305 proteins were generated using SomaScan v 1.3K (SomaLogic, Boulder, Colorado). Description of the SomaScan 1.3K assay is further described in^15^. Normalization follow SomaLogic’s guidelines for data processing encompass three sequential levels of normalization, namely Hybridization Control Normalization (Hyb) followed by Median Signal Normalization (Hyb.MedNorm) and Interplate Calibration (Hyb.MedNorm.Cal). There are no missing data on the platform. SomaScan 5K v4.0 (4776 proteins) was performed by SomaLogic and we used Adaptive Normalization by Maximum Likelihood (anmlSMP). For pQTL discovery, we used a rank-based inverse normal transformation to align protein levels to a normal distribution; however, for estimating genotype probabilities and associations with smoking, we used log transformed protein values.

### Statistical analyses

#### pQTL discovery by protein wide association study (pWAS)

**Figure 1:**
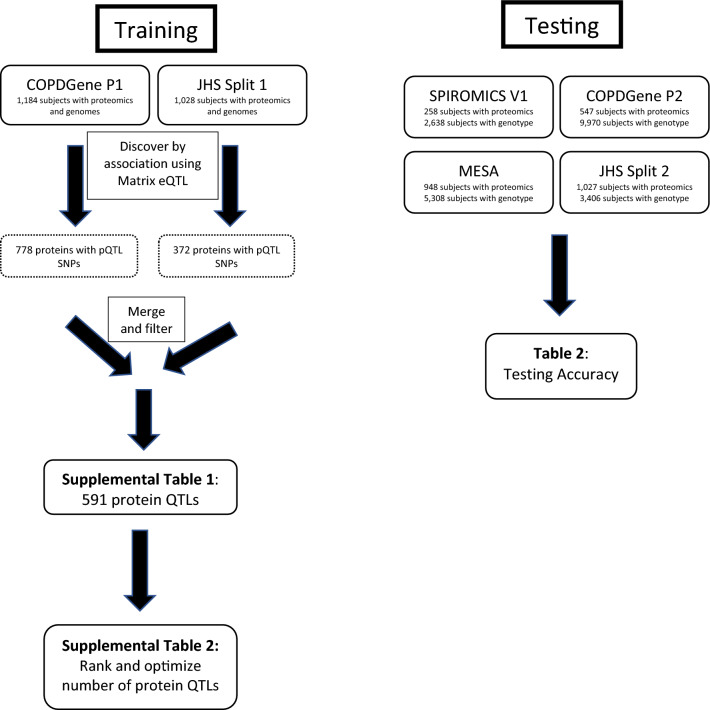
Strategy for identifying protein-QTL SNP combinations (training) and testing accuracy of proteins for identifying the subject by association with genotypes file.

COPDGene had genotyping for 691,764 SNPs without imputation. Genotype for these SNPs in JHS were called using TOPMed whole genome sequence. Only SNPs with minor allele frequencies (MAF) greater than 5% in the sample population were included for analysis. Both datasets were aligned to GRCh38. SNP-by-proteins associations were assessed in separately in both the COPDGene and JHS discovery cohorts using linear regression assuming an additive model by genotype. Analysis was performed using the R package ‘MatrixEQTL’ (version 2.2)^16^. Each model assessed direct association between protein level and genotype, with no adjustment for covariates. Protein quantitative trait loci (pQTLs) were considered significant at FDR corrected p-value < 0.05. The pQTL assessments in JHS and COPDGene were performed independently. After merging the two sets of pQTLs from the two training cohorts, we reduced the set to obtain a list of uniquely associated protein and SNP combinations. For each unique protein in the pQTL set, we kept only the highest significance SNP pQTL as determined by the p-value for the training cohorts (Fig. 1). When the two training cohorts had different top SNPs (often in linkage disequilibrium), we chose the SNP from the cohort with the lowest p-value. This first-level reduction produces a set of unique proteins, but in some cases, multiple proteins may be associated with the same SNP. If a SNP was associated with multiple proteins, we used only the protein with the highest protein association for that SNP. This process ensured that each protein and each SNP appear only once in our pQTL sets.

#### Bayesian modeling

**Figure 2:**
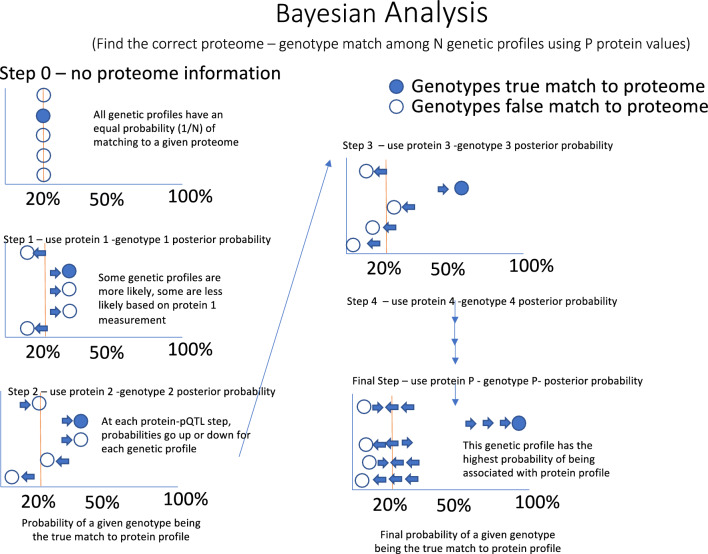
Naïve Bayes approach to estimate posterior probability of a subject matching genotype predicted by protein levels.

**Figure 3:**
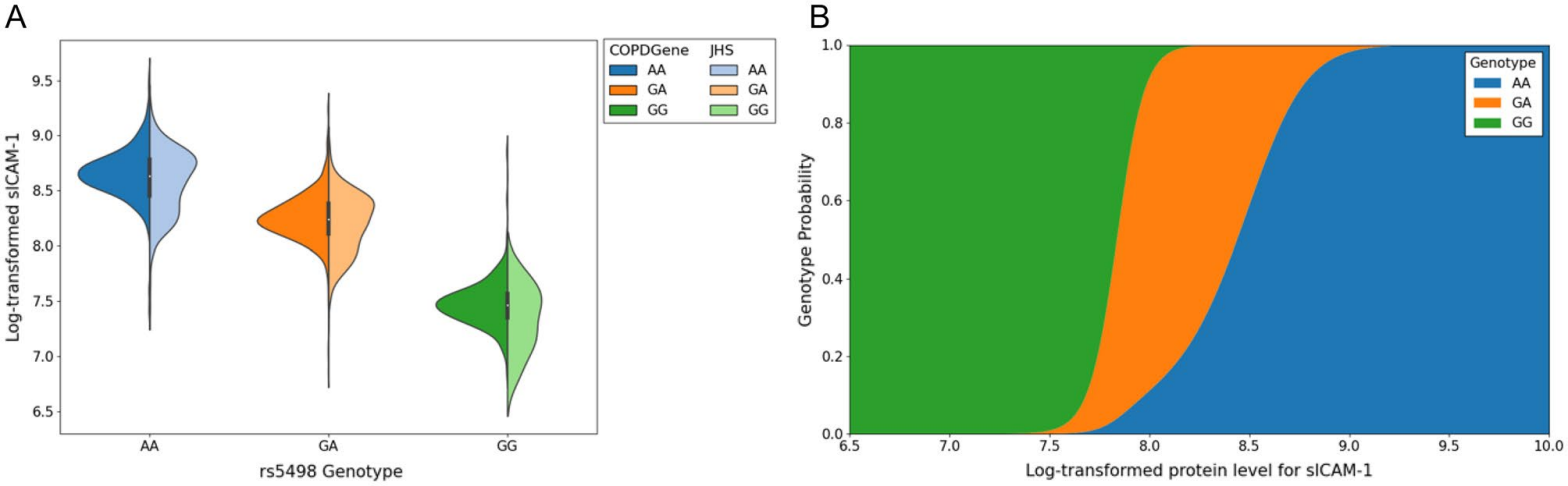
(**A**) Beeswarm showing the protein distributions for sICAM-1, which have been log transformed and stratified by genotype in COPDGene and JHS training sets. In this example AA is the major genotype. (**B**) Probability function for genotype by protein value for sICAM-1.

For predicting the probability of genome matching we use a Naïve Bayesian method (Fig. 2) which estimates the probability of observing genotype vector *g* using the genotype specific mean (µ) and standard deviation (σ) estimated from training data. This is similar to an approach used in genotype estimation from eQTLs^6^. To combine the training estimates from COPDGene and JHS we used the GaussianNB model from scikit-learn (version 0.23.2) for this estimation^6^. During training, we use the partial_fit method to calculate µ and σ parameters on a single dataset. The same method can be used to update parameters µ and σ, allowing us to train a model on multiple datasets by sharing the trained model. Since each SNP is biallelic, we calculate three probabilities corresponding to the three possible genotypes.

$$P(g|x) \propto P(g) \cdot P(x|g)$$

using a Gaussian naïve Bayes framework, where we define three normal probability distribution functions

$$P(x|g) = \frac{1}{{\sqrt {2\pi } \sigma_{g} }}e^{{ - \frac{{\left( {\frac{{x - \mu_{g} }}{{\sigma_{g} }}} \right)^{2} }}{2}}}$$

which describe the distribution of protein levels for each of the three genotypes (Fig. 3a), where μ_g_ and σ_g_ are the estimated mean and variance respectively of the protein levels *x* for subjects with genotype *g*. Under the naïve Bayes framework, we estimate the probability of the subject possessing each of the three genotype classes, given an observed protein level (Fig. 3b). By repeating this process for each of the *N* protein/SNP pairs, we obtain the probability of each genotype class for the top 100 SNPs. We calculate the odds of each genotype being the true genotype, and then using the known genotype values *g*_1_…*g*_*N*_ for each subject, we can compute the odds of observing the correct or “true” genotype vector ***g***^***true***^ for a subject as the product of the odds of observing the individual true genotype values.

$$Odds\left( {{\varvec{g}}^{{{\varvec{true}}}} {|}{\varvec{x}}^{{{\varvec{true}}}} } \right) = \mathop \prod \limits_{i = 1}^{N} \frac{{P(g_{i}^{true} |x_{i}^{true} )}}{{1 - P(g_{i}^{true} |x_{i}^{true} )}}$$

For each subject with proteome data, we calculate the odds of the genotype vector of every genotyped subject in the dataset. Assuming one of the genotyped subjects within the dataset is the true identity *S*_*true*_ with observed protein levels ***x***_***true***_ we take the genotype with the highest odds given the observed protein values as the “match” for this subject. If the genotype with the highest odds of match (top 1) belongs to the subject whose protein levels were observed, we consider this a match. We also tested whether the true match was among the three highest odds (top 3) and 1% highest odds (in top 1%).

#### Associations with smoking

A T-test was used to assess whether proteins (log transformed) were associated with current smoking (smoking cigarettes in the past 30 days).

#### Software and packages

All analyses were run in R (version 3.6.11) and Python (version 3.7). The code used in this manuscript is available on GitHub (https://github.com/BowlerLab/reidentify_code).

## Results

### Model training and parameter optimization

**Figure 4:**
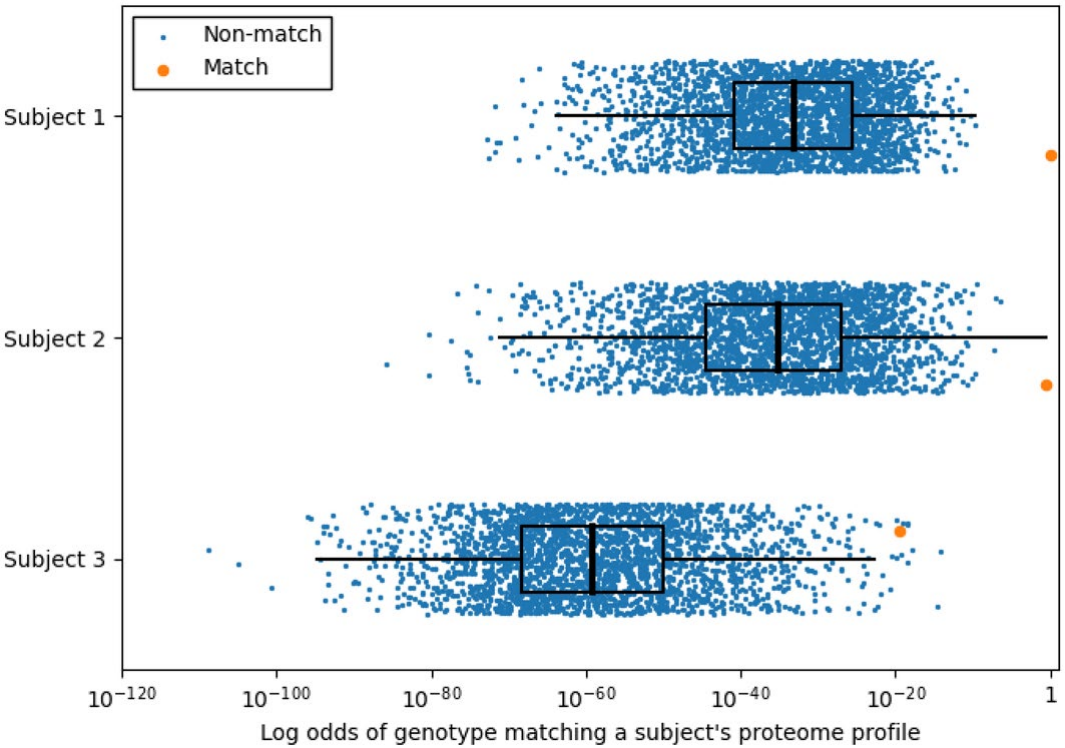
Probability that a proteome matches a given genome in the test dataset. In this example, 100 proteins are used to identify probable genotype at 100 pQTL SNPs. Most proteome profiles were associated with the correct genotype profile (orange circle) with near 100% probability of being correctly linked (Subject 1 and 2). The rest of the proteome profiles typically were represented in the top 1% of highest probability genotypes matches (top 26 of 2698) as demonstrated by Subject 3. The blue circles probability of genotype profile matching from incorrect subjects. The box plots show the 25–75 percentile range with the median and the whiskers represent 1.5 interquartile distance. The X-axis is plotted on a log scale.

**Figure 5:**
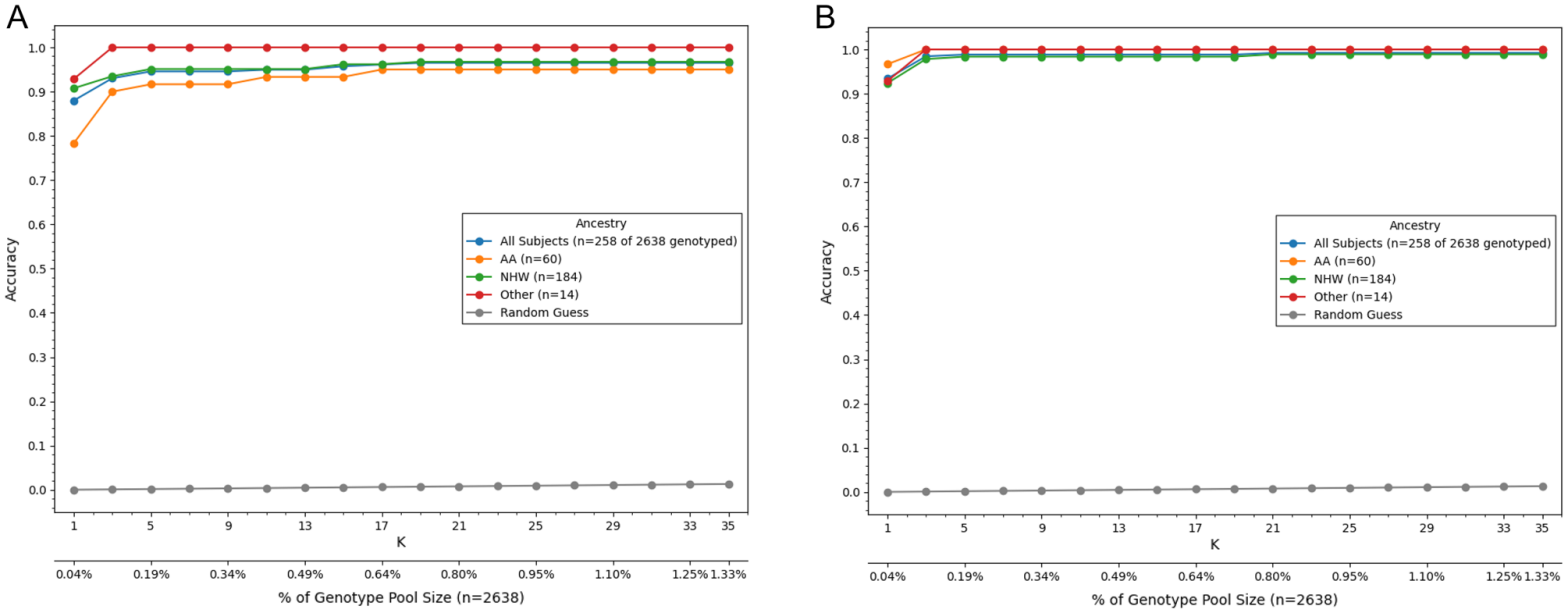
Training with data from diverse populations improves testing accuracy in African Americans (AA). (**A**) First attempts at training with only 13% AA subjects in SPIROMICS resulted in lower testing accuracy in independent AA compared to non-Hispanic White (NHW) subjects. (**B**) After training with both COPDGene and JHS subjects, identification accuracy significantly improved in AA subjects and similar to that of other races.

Our first training attempts at model training used only COPDGene subjects, which were mostly subjects with predominant European ancestry. This analysis identified 778 proteins with at least one pQTL SNP. To test the accuracy of protein measurements to predict genotypes, every proteome was assigned a probability of proteome matching genome (Fig. 4). The accuracy of the method was determined by how many times a subject with a proteome had the true genome assigned the highest probability of a match as the first choice, top three choices, or top 1% of the dataset. This method demonstrated excellent testing accuracy in identifying independent subjects of European ancestry in COPDGene, MESA, and SPIROMICS (83–92%); however, testing accuracy in subjects with predominantly African ancestry was significantly lower (61–76%). Therefore, we retrained our models using additional African-Ancestry subjects from JHS subjects. In the JHS training data set we identified 372 proteins with at least one pQTL SNP. We then combined the COPDGene and JHS training pQTLs for a total of 591 proteins with at least one pQTL SNP (Supplemental File 1). Using these combined COPDGene and JHS training set we significantly improved the matching accuracy in African American subjects (Fig. 5) which improved accuracy to ~ 90%, which is similar to accuracy in European ancestry subjects.

Next, we sought to determine the minimum number of protein-pQTL pairs that were necessary to match a proteome to a genome. First, we ranked protein-pQTL pairs by p-value and then retested using only smaller subsets of the strongest protein-pQTL pairs (Supplemental Table 1). Using the 1.3K assay overall accuracy plateaued at around 100 of the most significant protein-pQTLs pairs but including all nominally significant protein-pQTLs pairs led to slightly lower accuracy, suggesting that these lower significance pairs were introducing more noise than signal and accuracy and having additional protein information is not informative for matching to genomes.

### Testing accuracy of matching proteome to genome across diverse, independent cohorts

Using the top 100 protein-pQTL SNPs from the training data using (COPDGene and JHS training subjects), we then tested prediction accuracies in 4 cohorts (SPIROMICS, MESA, JHS, COPDGene) using independent subjects that had not been used for training, including accuracies based on race and ethnicity (Table 2). The true match was among the highest odds for most subjects (> 85%) in the cohorts and populations, except for COPDGene and Black Americans in MESA. If we took the top 1% of highest odds, the true match was among the highest odds for most subjects (> 85%) in all cohorts and populations.

**Table 2:** Accuracy of matching proteome profiles to genetic profiles using 150 proteins from SomaScan 1.3K data.

Testing Cohort	Subgroup	% correctly identified		
		Top 1 (%)	In top 3 (%)	In Top 1% (%)
COPDGene	Overall	85.0	89.0	97.8
	NHW	86.0	89.6	98.4
	AFA	75.5	83.7	91.8
JHS	AFA	85.8	91.5	98.1
MESA	NHW	97.3	98.5	99.5
	AFA	87.9	91.2	96.7
	Chinese-American	98.6	100	100
	Hispanic	97.2	99.7	99.7
SPIROMICS	Overall	93.4	98.5	99.2
	NHW	92.4	97.8	98.9
	AFA	96.7	100	100
	Other	92.9	100	100

To determine whether newer and larger proteome assays were more or less accurate at identifying genetic profiles, we randomly split 5292 COPDGene subjects (71% NHW and 29% AA) who had SomaScan v4.0 5K data (4776 proteins) into training and testing groups using a 50/50 train-test split (Supplemental Table 2) to generate a new list of protein-pQTL pairs (Supplemental File 2). We also used these novel protein-pQTL pairs to match 11,761 proteomes (8987 NHW and 2774 AA subjects) with 12,219 genomes (9345 NHW and 2874 AA subjects) and from the ARIC cohort. With as few as 100 proteins, identification accuracy improved to > 99% (Table 3) and accuracy in subjects with African ancestry was similar to those with predominantly European ancestry although accuracy was still slightly higher in European Ancestry compared to African Ancestry subjects (99% versus 98%). Accuracy was similarly > 98% in ARIC, even when > 92% genotype imputation was needed in ARIC. Adding additional protein-pQTL information beyond the top 150 tended to slightly decrease accuracy, most likely due to additional noise.

**Table 3:** Training and testing accuracy of matching proteome to genome for SomaScan 5K data.

Cohort	Training						Testing											
	COPDGene (N = 2646 genomes)						COPDGene (N = 9970 genomes)						ARIC (N = 12,219 genomes)					
Ancestry	European American (N = 1877 proteomes)			African American (N = 769 proteomes)			European American (N = 1870 proteomes)			African American (N = 776 proteomes)			European American (N = 8987 proteomes)			African American (N = 2774 proteomes)		
# Proteins	Top 1 (%)	In top 3 (%)	In top 1% (%)	Top 1 (%)	In top 3 (%)	In top 1% (%)	Top 1 (%)	In top 3 (%)	In top 1% (%)	Top 1 (%)	In top 3 (%)	In top 1% (%)	Top 1 (%)	In top 3 (%)	In top 1% (%)	Top 1 (%)	In top 3 (%)	In top 1% (%)
20	85.56	93.61	99.15	60.73	76.20	96.62	83.90	92.09	98.66	60.05	77.32	97.55	52.77	70.54	96.44	35.63	52.34	80.52
40	99.04	99.63	99.89	94.93	97.66	99.48	97.97	98.93	99.63	94.59	97.68	99.74	94.08	97.28	99.71	86.87	94.41	99.24
60	99.52	99.79	99.89	97.92	98.83	99.48	98.72	99.30	99.63	97.29	98.84	99.74	97.36	98.88	99.78	94.27	97.56	99.76
100	99.79	99.89	99.89	98.83	99.09	99.48	99.36	99.52	99.63	98.45	99.23	99.74	98.83	99.49	99.80	96.75	98.81	99.95
150	99.84	99.89	99.89	99.09	99.22	99.48	99.47	99.63	99.63	98.84	99.48	99.87	99.05	99.53	99.80	97.61	98.90	99.86
All	96.27	96.86	98.61	98.83	99.22	99.61	97.97	98.93	99.63	94.59	97.68	99.74	99.02	99.63	99.80	97.13	98.71	99.81

Using the same proteins described above, we show that we can identify individuals even without genetic databases using either the SomaScan 5K (COPDGene) or 7K (SPIROMICS) data. We show this by calculating Euclidean distances in N-dimensional space and show that this distance is the shortest for the same subjects over years compared to unrelated individuals (Supplemental Fig. 1). This demonstrates that the proteome by itself is mostly closely related to the proteome of the same across time. In the JHS cohort there were 314 subjects with proteome profiles and first-degree relatives in the genomic dataset. Among those 125 (39.8%) had at least 1 sibling in the top 1% of matches and 85 folks (27.1%) had all siblings in the top 1% of matches (Supplemental Fig. 2). This demonstrates that a proteome can help identify first degree relatives.

### Genome privacy protection through proteome transformation

**Figure 6:**
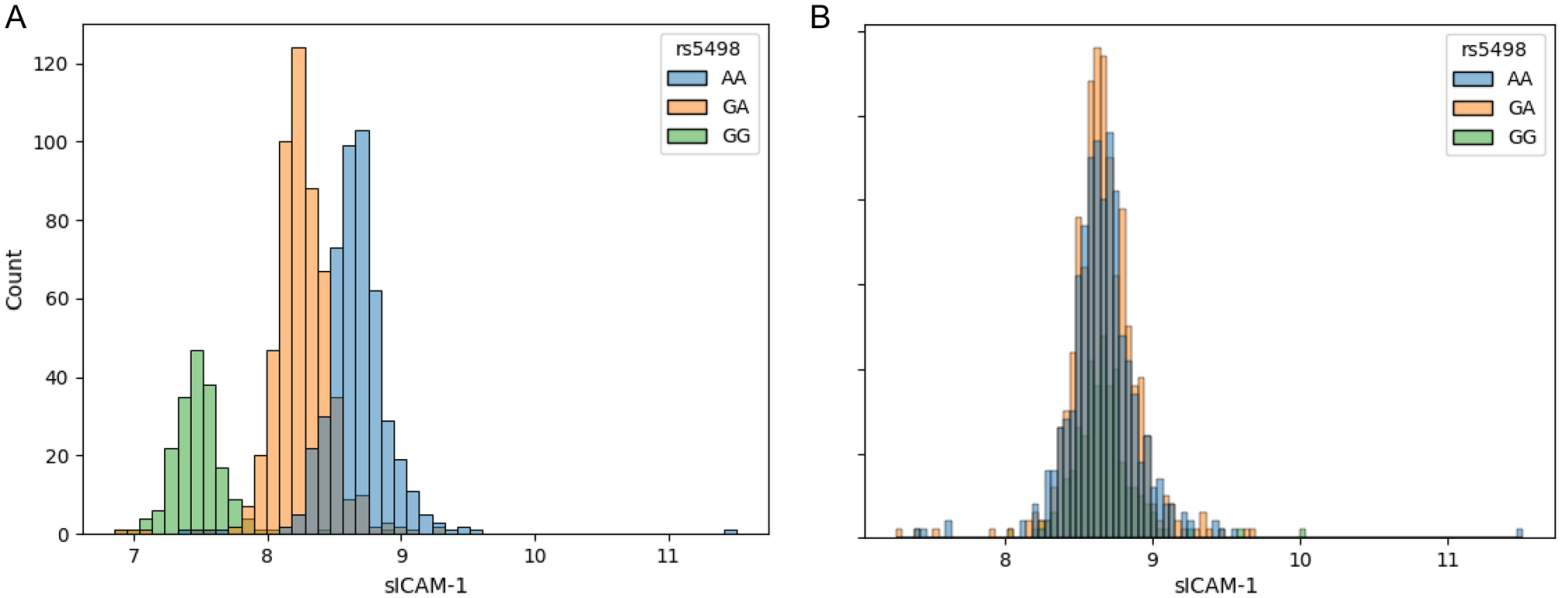
Poisoning data by adjusting protein values for genotype. (**A**) sICAM histograms showing normal probability distribution functions for sICAM-1, which have been log transformed. In this example AA is the major genotype. (**B**) Adjusting protein levels by recentering the mean on each genotype group abolishes the genotype effect on sICAM-1 measurements.

**Figure 7:**
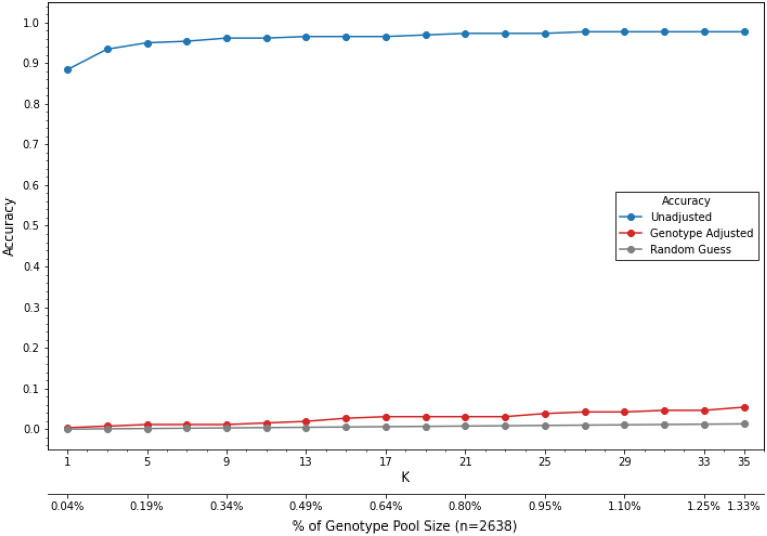
Removing the mean protein-pQTL effect abolishes the ability of matching a proteome to a genome. Shown are accuracy of matching algorithm with (red) and without (blue) removing mean pQTL effect as well as the probably of a random guess matching (grey).

Since we have shown that measurement of selected proteins with strong pQTLs can provide genetic information similar to a SNP, we reasoned that removing the pQTL effects on the proteome would inhibit the ability to reidentify a subject. One method that accomplishes this is to adjust each protein measurement by subtracting the population mean for that genotype (Fig. 6). This method has the advantage in that if the subject’s genotype and the correction factors are known, it is simple to recapitulate the actual protein measurements. In both testing cohorts, subtracting the genotype effect abolished the ability to identify subjects (Fig. 7).

### Can genotype adjustment preserve biomarker-phenotype associations?

**Figure 8:**
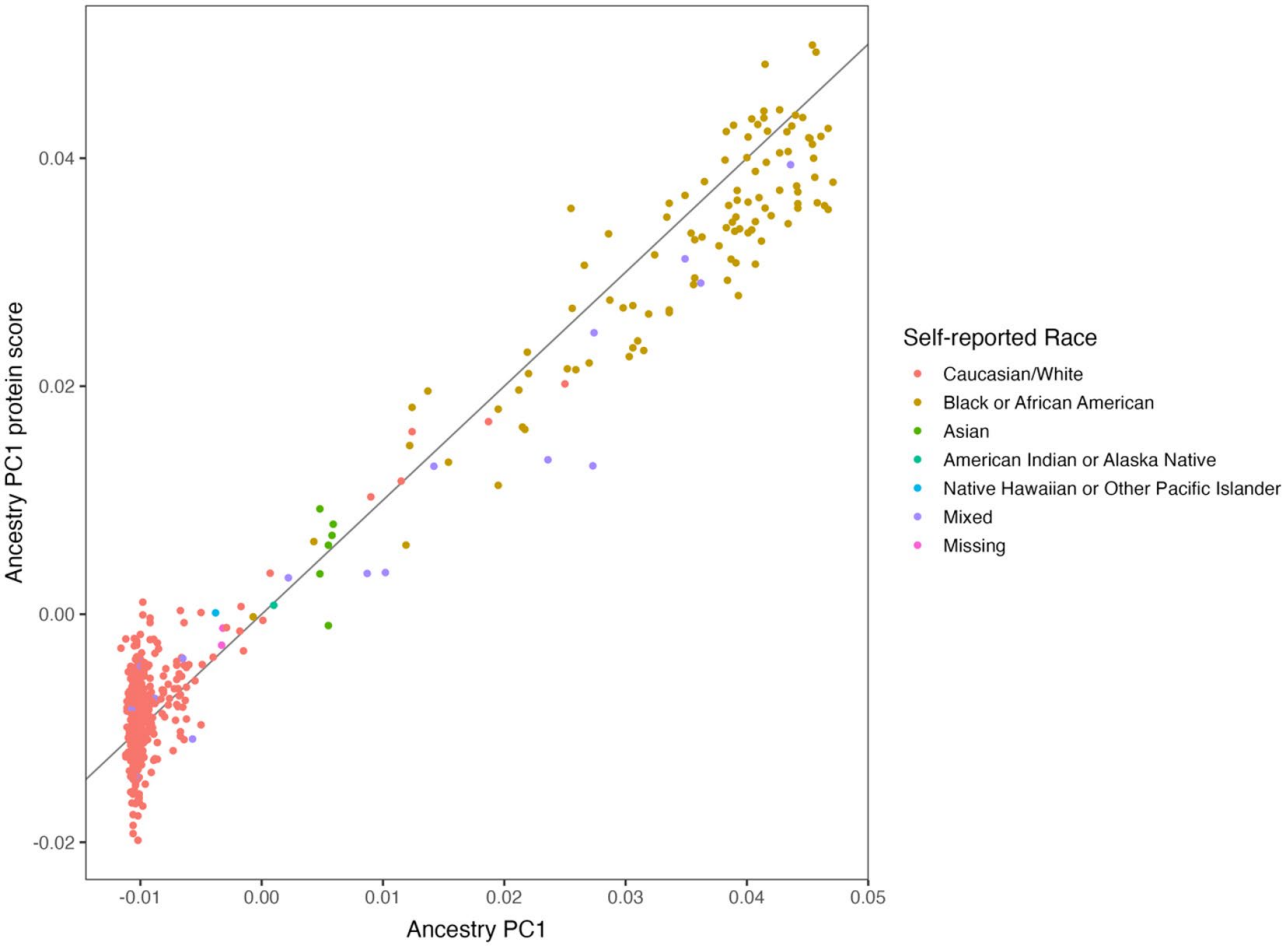
The proteome can accurately predict the percentage of genetic African Ancestry. In SPIROMICS, pooled genetic ancestry was calculated using genotypes as described (PMCID: PMC6090900). Using SomaScan 7K data we used elastic net to create an Ancestry PC1 (African ancestry) protein score and then used independent subjects to determine the correlation between the percent genetic African ancestry with protein ancestry. The correlation between protein ancestry score and genetic ancestry score was 0.98.

To test if adjusting for genotype affects associations between biomarkers and phenotypes, we first identified two proteins, sICAM-5 and DERM, which were significantly associated with smoking status in both the COPDGene and SPIROMICS testing cohorts. Next, we assessed the association before and after adjustment for genotype. In both cohorts, associations with smoking status did not change significantly after genotype adjustment (Supplemental Table 3). Using logistic elastic net we are also able to demonstrate that using 67 proteins from COPDGene 5K data, one can predict sex with > 99% sensitivity and specificity (Supplemental File 3 and 4). In SPIROMICS subjects we can also use elastic net to identify self-reported African American race and percent genetic African Ancestry (Fig. 8). The correlation between protein ancestry score and genetic ancestry score was 0.98.

### Using the matching algorithm to identify mislabeled samples in existing datasets.

**Figure 9:**
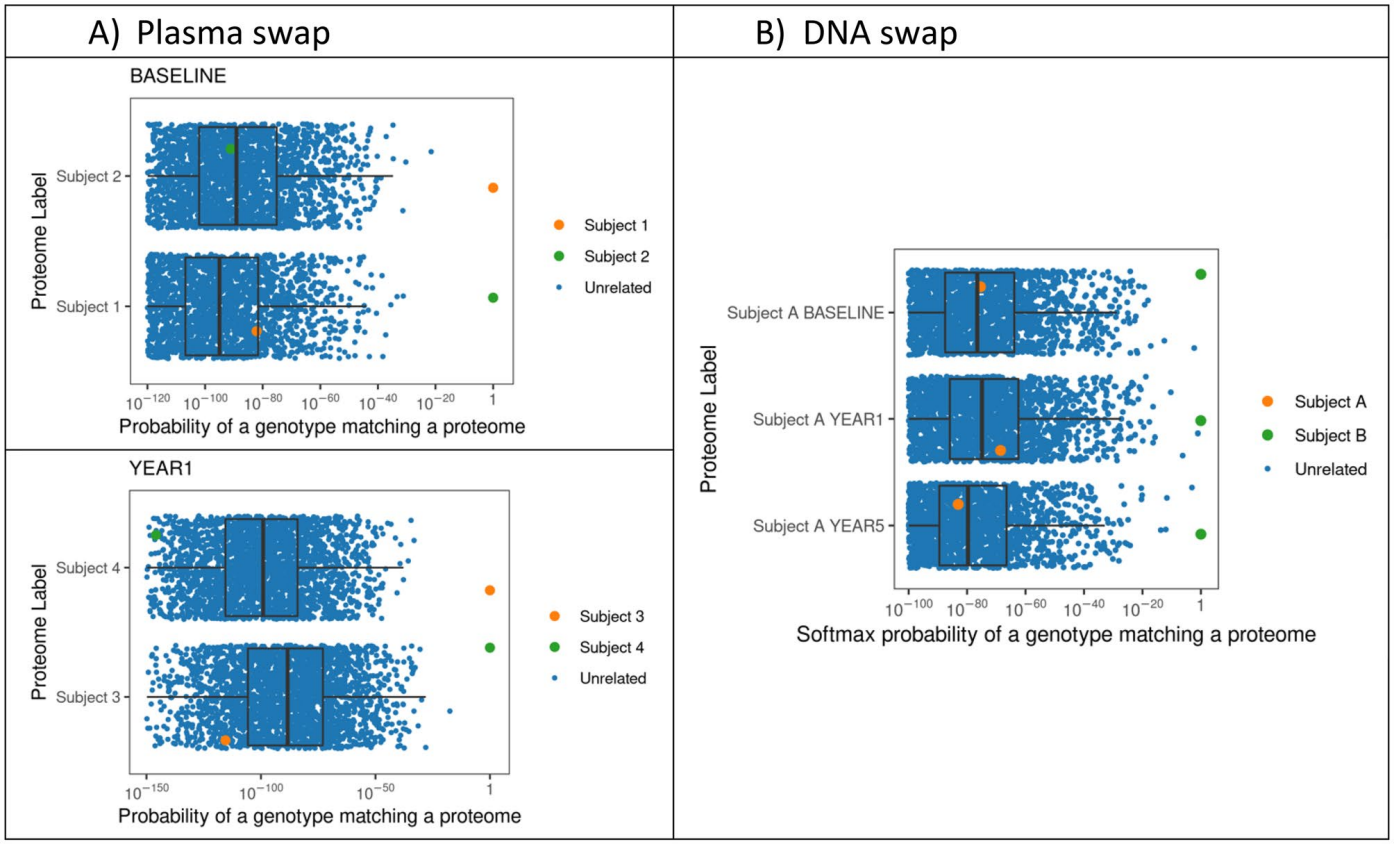
How the matching technique can be used to identified mislabeled omics data. (**A**) two subjects (1 and 2) were enrolled at the same clinical center at a baseline visit. Their plasma proteomes matched (P = 1) a different subject’s genome at baseline from the same clinical center, but their plasma proteomes matched the correct genome at subsequent visits. Another example of this is two subjects (3 and 4) from a different clinical center who appear to have their plasma samples swapped at their year 1 visit. This suggests that plasma samples were swapped at a single clinical center during a single visit and should be relabeled. (**B**) A subject (Subject A) who has multiple visits in which the proteomes were all mapping consistently to the genome of a different person (Subject B). This suggests that the DNA sample that was used for genotyping was swapped and that the DNA genotype data from Subject A should be labeled as coming from Subject B. Note that the x-axis for all the figures are shown on a log-scale because the probability all the unrelated a proteome matching to an unrelated genome is essentially zero (e.g., P < 10^40^).

In all our efforts to match proteomes with genomes, our matching accuracy seemed to plateau around 99.8%, even for the platforms with > 5000 proteins. In nearly all cases in which there was not a correct match of proteome to genome, the proteome had a nearly 100% probability of matching to a different genome. This suggests that either the proteome or genome has been mislabeled likely due to a swap of sample during the chain of custody from research subject to data generation. We assessed the extent and causes of poor matching by using SomaScan 7K data from SPIROMICS, in which of 18 of 5132 (0.2%) of proteomes did not exactly match their genome. In 8 of 18 proteomes the subject had multiple visits which generated proteomes, many of which matched to the same genome of a different person’s DNA, suggesting that the DNA was mislabeled and came from a different person. In 4 of 18 proteomes, all but one of the proteomes matched correctly to the genome and the mismatched proteome had a corresponding mismatched sample from the same visit. This suggests that a plasma sample was swapped between two subjects at a single visit (see examples Fig. 9). For 6 of 18 subjects who had mismatched genomes and proteomes, there was only one proteome and genome in the database and therefore we could not determine whether it was the proteome or genome that was mislabeled.

## Discussion

De-identification of data is a key concept for shared research and privacy protection but is not yet used in large scale proteomic studies. While small proof of concept studies have suggested that mass spectrometry can identify missense variants (minor allelic peptides) which can suggest specific SNPs^17^, this approach has not yet been used across large scale cohort studies nor with non-mass spectrometry proteomic data. This study is the first to demonstrate on a large scale that proteomic data are not identity protected because an individual proteome can be matched to a specific genome with high accuracy even without protein sequence information. The key identifying features in the proteome are the effects of common pQTLs, which link a measured protein level to a specific genotype. Furthermore, we show that identification only requires a small number of proteins (as few as 60–100 selected proteins) to link an individual protein profile to a single genetic profile among thousands of subjects and that it is accurate even with imputed genotypes. Additionally, our results suggest that using diverse subjects for selecting the most influential proteins improves overall accuracy, particularly among those with African ancestry and underscores the importance of including diverse subjects in Omics research. We show that proteomic data can identify behavioral features (e.g., smoking) even after removing the features that allow matching to genomes. The ability to accurately identify someone by linking their proteome to a genome, identify risk for protein related disease such as alpha-1 antitrypsin deficiency^18^, infer sex, genetic ancestry, or relatedness and also characterize other characteristics such as body fat, renal function, fitness, smoking, alcohol consumption, diabetes, cardiovascular risk^19^, and age^20^ implies that proteomic data should have at least the same (if not more rigorous) privacy protections as genetic and genomic datasets.

The two main technological breakthroughs that have facilitated accurately matching an individual proteome to a specific genome are improvement in high throughput proteomic technologies and large scale pQTL studies. Until the last few years, there were no proteomic platforms that could simultaneously and accurately measure more than 100 proteins and there was little known about which of those proteins had strong pQTLs. While our study used three different SomaScan platforms, lack of privacy (de-identification) should be implied for any platform that can simultaneously measure thousands of proteins even when mass spectrometry is not used. The logical continuation of this principle is that proteomic data could be used to discriminate based on identifying the sex of a subject, ancestry, or paternity. A protein profile could even be used to identify close relatives for forensic purposes.

The ability to link proteomes to genomes is not always a bad thing, particularly when cleaning data. For instance, we used matching to identify when genomes or proteomes are likely to have been mislabeled in large cohort databases. When more than 2 omics data sets are available from subjects, use of multiple pairwise matching can even pin-point which data entry is mislabeled. In our work we demonstrate examples of both plasma and DNA samples that are likely to have been swapped and have proposed corrections to the labeling of data. When used in a judicious manner, this matching technique can give confidence and improve the quality of multi-omic databases.

De-identification and privacy protection by informatics is a growing field. We acknowledge that our proposed privacy-preserving measures are only applicable when Naïve Bayes (NB) is used for profiling and we recognize the large body of emerging literature on alternative data obfuscation methods to protect privacy of many types of data^21^. These methods range from industry level data obfuscation/masking and secure data outsourcing techniques such as substitution, shuffling, numeric variance and null-out/mask-out, to more rigorous statistical data obfuscation methodologies used in Hippocratic Databases^22^, and privacy-preserving data mining^23^ such t-Closeness^24^, differential-privacy^25^ based methods. Machine learning^26^ and deep learning^27^ are also being used in proteomic feature identification and we may be able to leverage these same methods to isolate and "cloak" identifiable omics features while maintaining desirable statistical properties of the data for downstream application. We also believe new omics-specific privacy-preserving methods must be introduced to preserve privacy with omics data against model evasion attack methods that can target both traditional profiling models (such as NB) and modern deep learning-based profiling models.

Bioethicists had anticipated that other omics data such as proteomic data might one day be identifiable and create privacy concerns^28^ and our work demonstrates that this day has come even for proteomic technologies that do not rely on peptide sequencing. Unfortunately, most governmental policies do not yet apply to newer omics data such as proteomics (one exception may be the General Data Protection Regulation in the European Union, which protects biological equivalents of genotypes). We suggest biomedical research policies be clarified or amended to include any omics data (e.g., measurement of proteins or other molecules, such as metabolites) in which genotype can be ascertained^29^, but also that there be consideration beyond genotype equivalents to include all features of omics (e.g. behavioral information such as smoking). Because data protection is imperfect and frequently breached, a complementary solution to maintaining privacy might include bioinformatic and identity preserving adjustments to proteomic data. We demonstrated that adjusting out the genetic effects on protein measurements protects privacy by obfuscating the genetic effects, but it still does not change non-genetic associations (such as smoking). This strategy is simple and can be reversed, if necessary, when a researcher has the accompanying genetic information. A disadvantage to removing genetic coding of the proteome is that it could remove associations in which genotype mediates protein affect. Another caveat from our work is that if training the method does not include diverse populations, the identification methods may not be generalizable outside European ancestry. While lower identifiability may be beneficial, future privacy protection algorithms may suffer if identifying features in underserved population are not fully known.

## Supplementary Information

Supplementary Tables.

Supplementary Figures.

Supplementary Information 1.

Supplementary Information 2.

## Data Availability

*COPDGene.* Genotype data and SomaScan can be found on dbGaP for COPDGene (phs000179). *JHS*. Genotype data can be requested through TOPMed and SomaScan can be found on dbGaP (phs000964). *SPIROMICS*. Genotype data and SomaScan can be found on dbGaP (phs18817) or through contacting the SPIROMICS GIC (https://www.spiromics.org/spiromics/contact-gic). *MESA*. Genotype and SomaScan data can be requested through TOPMed and dbGaP (phs001416). *ARIC*. Individual genotyping data from ARIC are available via dbGaP (phs000668). Proteome data, as well as phenotypic data, are available via application through the ARIC Data Coordinating Center (https://sites.cscc.unc.edu/aric/distribution-agreements).
