# Supplementary material for: Large scale proteomic studies create novel privacy considerations
Source: Sci Rep. 2023 Jun 7;13:9254. doi: 10.1038/s41598-023-34866-6 (PMC10247808; doi:10.1038/s41598-023-34866-6)
Supplement: Supplementary file 1 — Supplementary Tables. [file 41598_2023_34866_MOESM1_ESM.docx]

**Supplemental Tables**

| **Supplemental Table 1**: Optimizing of number of training proteins | | | | | | |
| --- | --- | --- | --- | --- | --- | --- |
|  | COPDGene Training | | | JHS Training | | |
|  | % correctly identified subjects | | | % correctly identified subjects | | |
| # proteins | Top 1 | In top 3 | In top 1% | Top 1 | In top 3 | In top 1% |
| 20 | 68.7% | 79.8% | 95.3% | 53.8% | 73.2% | 93.6% |
| 40 | 82.5% | 89.7% | 96.8% | 79.9% | 89.0% | 97.8% |
| 60 | 88.1% | 93.2% | 97.5% | 85.7% | 92.3% | 98.4% |
| 100 | 92.1% | 94.8% | 97.7% | 91.1% | 95.1% | 99.3% |
| 150 | 94.3% | 96.5% | 98.1% | 93.2% | 96.7% | 99.6% |
| 250 | 93.0% | 95.1% | 97.8% | 92.5% | 96.8% | 99.7% |
| All | 91.0% | 93.3% | 96.2% | 92.3% | 96.4% | 99.6% |

| **Supplemental Table 2**: Characteristics of training cohort and independent testing cohorts with SomaScan 5k | | | |  |  |
| --- | --- | --- | --- | --- | --- |
|  | Training | Testing | |  |  |
| Cohort | COPDGene  (N = 2,646 with SomaScan  N = 2,646 genotyped) | COPDGene  (N = 2,646 with SomaScan  N = 9,970 genotyped) | ARIC  (N = 11,761 with SomaScan  N = 12,219 genotyped) |  |  |
| Gender (%female) | 50.6% | 48.4% | 55.4 |  |  |
| Age (± SD) | 65.3 ± 8.5 | 65.7 ± 8.7 | 57 ± 5.7 |  |  |
| Race/ethnicity (self-reported) |  |  |  |  |  |
| White, non-Hispanic | 70.9% | 70.7% | 75.6% |  |  |
| Black, non-Hispanic | 29.1% | 29.3% | 24.4% |  |  |
| SD – standard deviation; | | |  |  |  |

| **Supplemental Table 3:** Association with smoking status is preserved after adjusting for genetic effect. | | | | | | | | |
| --- | --- | --- | --- | --- | --- | --- | --- | --- |
|  |  | No Genotype Adjustment | | | Genotype Adjustment | | | |
| **Dataset** | **Protein** | **t-statistic** | **smoking difference** | **p** | **t-statistic** | **smoking difference** | **p** | |
| SPIROMICS | DERM | 5.64 | 0.181 | 6.0E-08 | 5.54 | 0.176 | 9.4E-08 | |
|  | sICAM-5 | -3.31 | -0.150 | 0.0012 | -3.33 | -0.137 | 0.0011 | |
| COPDGene | DERM | 9.86 | 0.139 | 5.5E-22 | 9.63 | 0.133 | 4.7E-21 | |
|  | sICAM-5 | -4.70 | -0.087 | 3.0E-06 | -5.45 | -0.092 | 6.5E-08 | |
| DERM – dermatopontin; sICAM-5: soluble Intracellular adhesion molecule 5; smoking difference the mean of current smokers – mean never and former smokers | | | | | | | |  |
