# Supplementary material for: Large scale proteomic studies create novel privacy considerations
Source: Sci Rep. 2023 Jun 7;13:9254. doi: 10.1038/s41598-023-34866-6 (PMC10247808; doi:10.1038/s41598-023-34866-6)
Supplement: Supplementary file 2 — Supplementary Figures. [file 41598_2023_34866_MOESM2_ESM.docx]

| 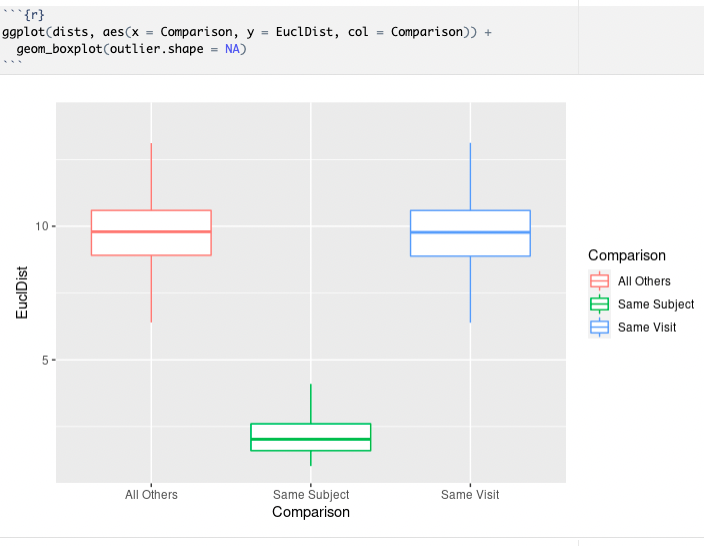 |
| --- |
| **Supplemental Figure 1**: **A proteome can be linked to another related proteome.** Using the top 100 protein-pQTLs, we mapped each proteome from COPDGene 5- and 10-year visits into 100-dimensional space and then calculated the Euclidean distance to all other proteomes. The closest proteome was nearly always a different proteome from the same subject at a different visit, demonstrating that we can map proteome-to-proteome without any specific genomes. |

| 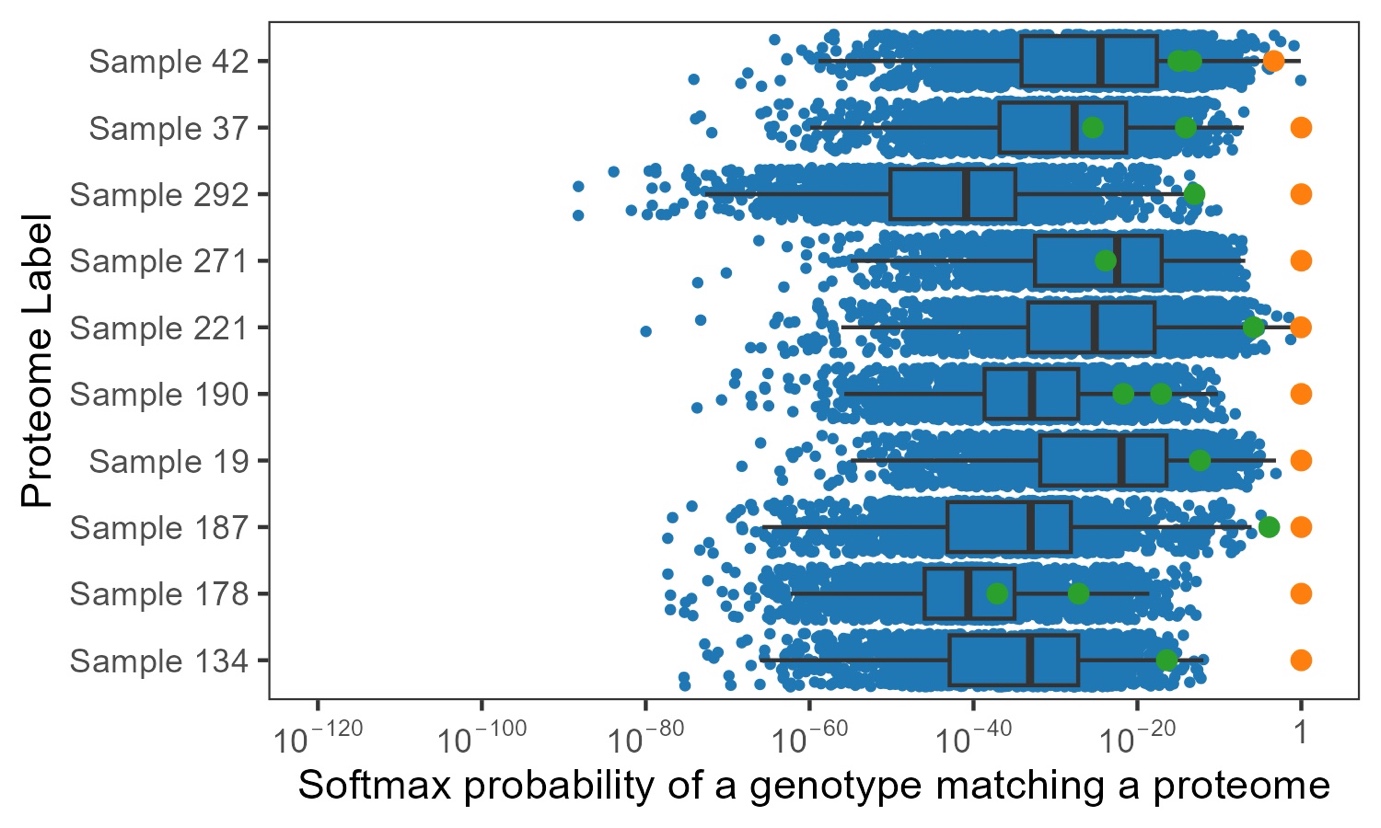 |
| --- |
| **Supplemental Figure 2**: **A proteome can be closely linked to genetically related individuals.** Using 10 individuals (orange circle) from JHS who had proteomes and first-degree relatives in the genome dataset (green circles), we demonstrate that a proteome can often be linked to a first degree relative (e.g., Subjects 292, 221, 187, 134). |
